# Acute Toxicity and Hazardous Concentrations of Zinc to Native Freshwater Organisms Under Different pH Values in China

**DOI:** 10.1007/s00128-018-2441-2

**Published:** 2018-09-24

**Authors:** X. F. Li, P. F. Wang, C. L. Feng, D. Q. Liu, J. K. Chen, F. C. Wu

**Affiliations:** 1 College of Environment, Hohai University, Nanjing, China; 2 State Key Laboratory of Environmental Criteria and Risk Assessment, Chinese Research Academy of Environmental Sciences, Beijing, China; 3 Faculty of Environmental Science and Engineering, Kunming University of Science and Technology, Kunming, China

**Keywords:** Zinc, Native aquatic species, Hazardous concentration, pH, Water quality criteria, Metal bioavailability

## Abstract

Zinc bioavailability to aquatic organisms varies greatly under different pH values. In the present study, five native species in China and four common international test species were selected to investigate the influence of changing pH on acute zinc toxicity. The results showed that the higher trophic levels exhibited increasing sensitivity to zinc as pH decreased. However, when the pH value was between 8 and 11, the acute toxicity of zinc was relatively constant. In addition, by using a species-sensitivity distribution (SSD) method, the short-term hazardous concentrations of zinc at different pH values (based on the 5th percentiles of the pH-specific SSDs) were determined to be 17.26 µg/L (pH 4), 48.31 µg/L (pH 5), 80.34 µg/L (pH 6) and 230.6 µg/L (pH 7), respectively. The present study provides useful information for deriving water quality criteria and assessing the risks of metals in the near future.

**Electronic supplementary material:**

The online version of this article (10.1007/s00128-018-2441-2) contains supplementary material, which is available to authorized users.

Zinc is widely distributed throughout the natural environment and is also an essential trace element for aquatic organisms and humans (Wang et al. 2000). When the dose of zinc exceeds a certain amount, it may cause adverse effects to the organisms (Lindholmer 1974; Smirnova and MelNichenko 1997; Wadige et al. 2017; Wu et al. 2011). According to the water quality criteria for zinc issued by the US EPA (2009), the criteria of zinc to protect freshwater aquatic organisms are 120 µg/L (short -term hazardous concentration) and 120 µg/L (long-term hazardous concentration), while the criteria for human health are 7400 µg/L (ingested potable water + edible aquatic organisms grown in the water) and 26,000 µg/L (edible aquatic organisms only). It is evident that aquatic organisms are much more sensitive to zinc concentrations in water than humans. According to the statistics of the National Bureau of Statistics of China in 2016, China is the second largest country in the world with zinc reserves, extraction, import and consumption (Guo and Feng 2013). Fu et al. (2016) reported that concentrations of zinc in Tai Lake were detected to be 0.018–1.246 mg/L, and approximately 50.7% of the aquatic organisms were predicted to be affected by zinc. In Songhua River in China, concentrations of zinc were from 0.92 to 70.81 mg/L (Liu et al. 2017). Therefore, the exposure risk to aquatic organisms due to zinc greatly exceeds that of other countries. Thus, the potential toxicity of zinc to aquatic organisms in China should receive more attention (Wu et al. 2011, 2012).

Presently, much research has been conducted on aquatic toxicity effects of zinc. For example, the main research direction of European and American countries focused on the toxic effects on Salmonid fishes, shrimp, flea, hydroids and phytoplankton genera (Alsop and Wood 1999; Diamantino et al. 2003; Heijerick et al. 2003; Karntanut and Pascoe 2002; Mottin et al. 2010; Muyssen and Janssen 2007; Nor 1990; Ryan et al. 2009). Due to the differences in the aquatic species, quantity, nutritional structure and other characteristics between China and European and American areas, Chinese investigators further studied the toxic effects of zinc on common Cyprinid fishes and other species exhibiting regional characteristics of China and Asia (Feng et al. 2013; Wu et al. 2011, 2012). However, most research studies only considered the zinc toxicity effect on single species such as fish, benthic animals or phytoplankton. Further, the influences of variations in natural water environmental factors on zinc toxicity were not considered.

The toxicity of metals is significantly influenced by physical and chemical parameters of water quality such as hardness, pH, and temperature (Alsop and Wood 1999; Clifford and McGeer 2009; Ryan et al. 2009; Zhao et al. 2010). The pH values of the rivers and lakes in different regions of China are very different, and seasonal changes are also obvious (Fu et al. 2016). There are also significant differences in the toxicity of metals in various water bodies. Some of the lakes or rivers may be acidic (pH < 6) or alkaline (pH > 9) for a short time if they were polluted by industrial wastewater or influenced by algae blooms (You et al. 2007; Yu et al. 2013). However, most of the current studies were carried out when the pH was in the range of 6–8, and they rarely addressed changes in the toxicity effect of zinc on aquatic species in acidic and basic water bodies (US EPA 2007). At the same time, the criteria and standard values established by each country, according to toxicological data, were created under conditions when the pH values of different water bodies were in the range of 6–8, which is not in accord with the actual situations of water bodies.

To address influence of the aforementioned physico-chemical water quality parameters on metal toxicity, a biotic ligand model (BLM) was developed to predict the toxicity of metals in the environment. The model comprehensively considers various water factors and action for the free metal ion bio-ligands (And and Wood 2004; Clifford and McGeer 2009; Heijerick et al. 2003; Ryan et al. 2009; Schamphelaere et al. 2005). The US and the EU BLM models for zinc are able to make predictions for safe concentrations based on test data for salmonid and warm-water fishes, invertebrates, and aquatic plants. However, almost all of the extensive data are restricted to North American and European temperate aquatic species tests, so there are still some questions with regard to the applicability of these BLMs to predict the sensitivity of the native species in China (Feng et al. 2013; Wang et al. 2006; Zhang et al. 2017). At the same time, judging from the accuracy of the predictions of the BLM, even if the responses of North American species are predicted, the predicted result is not very satisfactory (Chen et al. 2010).

Therefore, in the present study, five native species in China and four common international test species were chosen as test organisms to elucidate the effects of pH on zinc toxicity in a wide range of pH values (3–11). The aims of the study were to (1) explore the changes in the zinc toxicity effect on the aquatic organisms in environments with different pH values and (2) compare the short-term hazardous concentration differences under different pH values with the current acute water quality criteria for zinc. Further, the results might provide an important part of the scientific basis for the establishment and management of water quality criteria or standards for zinc and other metals under complicated water quality conditions.

## Materials and Methods

The reagent ZnSO_4_·7H_2_O with 99% purity was purchased from the Sigma-Aldrich Chemical Company in the USA. The laboratory uses various sources of natural waters to mix with deionized water for at least 24 h to prepare standard dilution water according to OECD standards (1992, 2004, 2011). The physical and chemical parameters of the standard diluted water are pH = 7.8 ± 0.2; dissolved oxygen concentration above 80%; hardness of 250 ± 25 mg/L (calculated by CaCO_3_); and ρ(Ca)/ρ(Mg) of approximately 4:1.

In this experiment, eight aquatic animals and one kind of aquatic plant, which are commonly observed in China, including *Pseudorasbora parva, Misgurnus anguillicaudatus, Macrobrachium nipponense, Cipangopaludina cathayensis, Bufo gargarizans, Limnodrilus hoffmeisteri, Daphnia magna, Scenedesmus obliquus* and *Chironomus riparius*, have been chosen as the experimental objects to conduct acute toxicity experiments. Among these species, *Scenedesmus obliquus* belongs to the first trophic level; *Chironomus riparius, Limnodrilus hoffmeisteri* and *Daphnia magna* belong to the second trophic level; *Pseudorasbora parva, Misgurnus anguillicaudatus, Macrobrachium nipponense, Cipangopaludina cathayensis* and *Bufo gargarizans* belong to the third trophic level. The selected species could represent different trophic levels, and also meet the species needs of water quality criteria research. The experimental species were acclimated for 1 week prior to the beginning of the experiment (OECD, ASTM 2014). The exposure experiments with *Pseudorasbora parva, Misgurnus anguillicaudatus, Macrobrachium nipponense, Cipangopaludina cathayensis*, and *Bufo gargarizans* were conducted under flow-through experimental conditions. Experiments using *Limnodrilus hoffmeisteri, Daphnia magna, Scenedesmus obliquus*, and *Chironomus riparius* were conducted as semi-static experiments, and the water was changed every 24 h. The illumination conditions during the experiment were light:dark times of 12 h:12 h, and the temperature of the circulating water bath was controlled at 20.0 ± 1.0°C. Ten experimental organisms were placed in each experimental cylinder. Exposure gradients of eight zinc concentrations were set up for each species. Based on the toxicity data of zinc to aquatic organisms in previous studies, the zinc gradient range of all species corresponding was between 0.025 and 64 mg/L (Feng et al. 2013; Liu et al. 2017; Wu et al. 2011). The pH value of each concentration was adjusted by using HCl and NaOH. All the nominal and measured concentrations for zinc were listed in Table S1.

To avoid contamination from the gastrointestinal tract, species were starved for 24 h before the experiment. Temperature, pH, dissolved oxygen and the total zinc concentrations in the solution were measured every 24 h, and the state of the experimental animals was observed and recorded every 24 h. The number of deaths of the tested species was recorded every day, and the 96 h or 48 h half-lethal concentration (LC_50_) was calculated for the aquatic animals. But for *Scenedesmus obliquus*, half-effective concentration (EC50) is based on 96 h-growth inhibition. The inhibition of growth was expressed as logarithmic algal biomass increase during the exposure period. Growth and growth inhibition were quantified from measurements of the algal biomass density.

Three replicates and one blank control were set up for each species under each pH value and zinc concentration. One-way analysis of variance (ANOVA) was performed on the treatment and control groups. Probability values less than 0.05 were considered as statistically significant. Concentrations of zinc before and after the experiment were measured. The within-treatment standard deviation values were less than 10%. The mortality rates in the blank control groups were also less than 10%. Concentrations of dissolved zinc were analyzed by inductively coupled plasma-mass spectrometry (ICP-MS, Agilent 7500a, USA). The main operating parameters of the instrumentation were as follows: radio-frequency power was 1500 W; radio-frequency voltage was 1.85 V; carrier gas flow was 1.22 L/min; sampling depth was 6.0 mm. The limit of detection (LOD) of the instrumentation was 0.001 mg/L.

The hazardous concentration of zinc was derived using the species sensitivity distribution (SSD) method. SSD was a data distribution fitting method. In brief, SSD mainly included four steps, including selecting of toxicity data, calculation of cumulative probability, data fitting and hazardous concentration extrapolation (Feng et al. 2013; Ministry of Environmental Protection of the People’s Republic of China 2017; Wu et al. 2012). All the data were fitted by several models (e.g., logistic, normal, and value distribution). Finally, the estimated zinc concentration corresponding to the cumulative probability at the 5th percentile of the SSD curve was used to estimate the hazardous concentration of zinc.

## Results and Discussion

It was found through the experimental study that the mortality of different aquatic organisms at different pH and zinc levels could be divided into three different groups (i.e., pH values between 3 and 8, 8 and 10, and greater than 10). At the first group (pH range of 3–8), with the decrease in the pH value in the aquatic environment, the mortality of all species had risen significantly under the same zinc dose level. Meanwhile, the growth inhibition of algae also increased. At the second group (pH range of 8–10), even with relatively high zinc concentrations, the mortality of any of the nine test species did not rise above control levels (less than 5%). At the third group (pH greater than 10), the mortality of most species was close to 100%, even at very low dissolved zinc concentrations in the water.

At present, the general view of the mechanisms of metal toxicity is that metal ions could combine with biological macromolecules in organisms such as protein, thus changing their activity. (Amiard-Triquet et al. 1998; Geffard et al. 2005). When zinc accumulates to a certain degree in organisms, the metal ion rate of entering an organism exceeds the synthesis rate of the metal-binding protein. The redundant metal ions will interact with other biological molecules in the body including enzymes and nucleic acids, thus causing poisoning. When pH in water is in the range of 3–8 at the first stage and there is a decrease in pH, Zn^2+^ is easily absorbed and enriched by organisms because the main forms of zinc in water are changed from Zn(OH)_4_^2−^, Zn(OH)_3_^−^, Zn(OH)_2_ and Zn(OH)^+^ to the dissolved free ionic form of Zn^2+^, and the toxicity of the simple positive ion Zn^2+^ is far greater than other forms of zinc. Further, the acidic enhancement can damage the tissues and organs of animals, so their resistance to zinc toxicity will be reduced. Therefore, the zinc toxicity of various kinds of organisms will increase with the reduction in the pH value. When pH in water is in the range of 8–10 at the second stage, the zinc toxicity does not increase with the increase in the concentration because most of the organisms can adapt to the weak alkaline condition. The Zn^2+^ and OH^−^ mainly exist in the complexation form in the weak alkaline water, which greatly reduces the effective concentrations of the Zn^2+^ form in water. When the pH of the water is over ten, various kinds of organisms cannot adapt to the strong alkaline water. The mortality of various kinds of organisms is more than 95% even without zinc, which is obviously affected by the excessive hydroxide ion in water.

When the pH value of the water environment was seven, hydrogen ions had a rather small influence on the mortality of various organisms. All the 96-h LC_50_/EC_50_ values of the experimental species were acquired under the standard water conditions (Table 1). The rank of toxicity for of the nine test species to zinc was also obtained as follows: *Scenedesmus obliquus* > *Daphnia magna* > *Limnodrilus hoffmeistteri* > *Macrobrachium nipponensis* > *Cipangopaludina cathayensis* > *Chironomus riparius* > *Pseudorasbora parva* > *Bufo gargarizans* > *Misgurnus anguillicaudatus*. According to the above ranking, it can be easily determined that when the nutritional level of the lower-ranked aquatic organisms remained low, the toxicity effect of zinc was more clearly exhibited by the smaller LC_50_ value. The lower trophic level organisms can easily absorb zinc and enrich the organisms. The tissues and organs of the animals with higher nutrition are relatively developed. Their abilities to internally detoxify and excrete zinc are stronger than those of the aquatic organisms at the lower trophic level.

**Table 1:** Acute toxicity of zinc under different pH values (LC_50_/EC_50_) (n = 81)

pH values	LC_50_/EC_50_/(mg/L)								
	*Scenedesmus obliquus*	*Daphnia magna*	*Limnodrilus hoffmeisteri*	*Macrobrachium nipponense*	*Cipangopaludina cahayensis*	*Chironomus riparius*	*Pseudorasbora parva*	*Misgurnus anguillicaudatus*	*Bufo gargarizan*
3	–	–	–	–	–	–	–	–	–
4	0.057 ± 0.004	0.041 ± 0.003	0.11 ± 0.014	–	0.547 ± 0.058	1.59 ± 0.158	2.84 ± 0.301	6.61 ± 0.575	2.38 ± 0.216
5	0.121 ± 0.017	0.054 ± 0.005	0.13 ± 0.016	1.12 ± 0.132	0.841 ± 0.089	4.99 ± 0.518	9.47 ± 0.919	11.90 ± 1.314	6.68 ± 0.572
6	0.158 ± 0.021	0.101 ± 0.011	0.23 ± 0.022	2.50 ± 0.217	2.263 ± 0.215	9.57 ± 1.106	13.54 ± 1.466	23.75 ± 3.154	11.11 ± 1.26
7	0.217 ± 0.023	0.233 ± 0.026	0.40 ± 0.037	2.96 ± 0.304	4.76 ± 0.451	13.71 ± 1.163	26.15 ± 3.032	35.98 ± 4.095	32.02 ± 3.81
8–11	–	–	–	–	–	–	–	–	–

Data are expressed as mean ± SD. “–” indicates that the value cannot be obtained through the calculation method

**Fig. 1:**
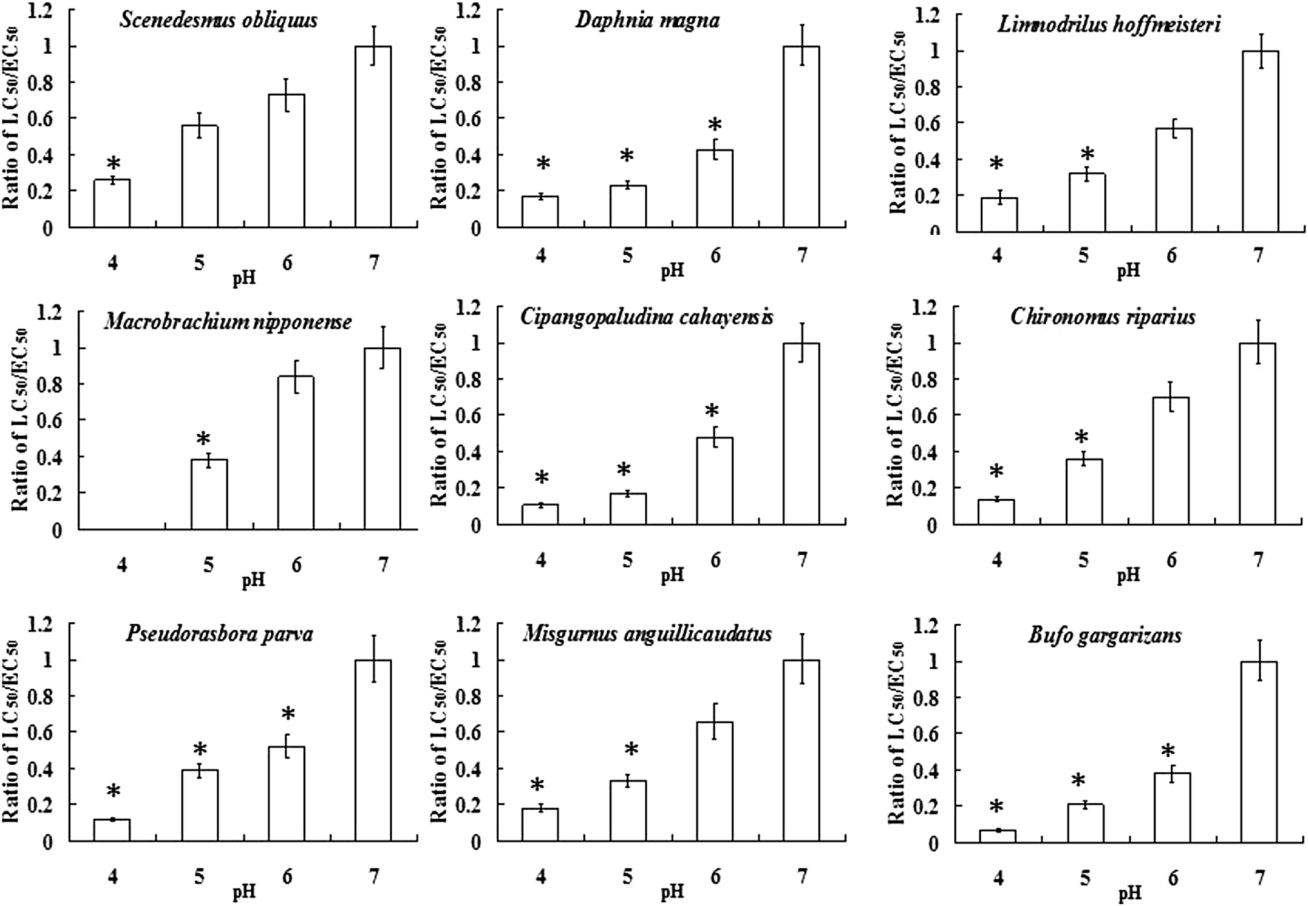
The sensitivity of the experimental species to zinc toxicity (pH 4–7). Data are expressed as mean ± SD; *p < 0.05

When pH was seven, hydrogen ions have a rather small influence on the mortality of various organisms. Thus, in this study, the LC_50_/EC_50_ values of pH equal to seven were selected as the standard values for various species. As mentioned above, with the reduction in pH from 7 to 4, zinc toxicity to various organisms had also increased. However, the degree of increase differed among the species. To quantify the degree of the pH influence on each species, all the LC_50_/EC_50_ values of pH equal to 4, 5 and 6 were divided by the above acquired standard value of LC_50_/EC_50_ values, and then, the ratios of each were acquired, which could indicate the changes in zinc toxicity sensitivity of various organisms when pH decreased (Fig. 1). Statistical analysis showed that there was significant difference for each species in the process of pH decreasing from 7 to 4. Moreover, significant differences among all the species were also observed. The toxicity effect of the reduction in pH on the lower trophic level aquatic organisms was not as obvious as that for the high trophic-level aquatic organisms such as fish or shrimp. The rank of various organisms’ relative changes in sensitivity to zinc was *Macrobrachium nipponensis* > *Bufo gargarizans* > *Cipangopaludina cathayensis* > *Pseudorasbora parva* > *Chironomus riparius* > *Daphnia magna* > *Misgurnus anguillicaudatus* > *Llimnodrilus hoffmeistteri* > *Scenedesmus obliquus*. The reason might be that the lower trophic level organisms have a stronger capacity to adapt to acidic and alkaline water. At the same time, the peracid and peralkaline water environment causes more obvious damage to the tissues and organs of animals with high nutrition such as gill, lungs, or blood, which could greatly reduce the degradation and excretion, and toxicity resistance abilities of high aquatic animals such as fish and shrimp.

**Fig. 2:**
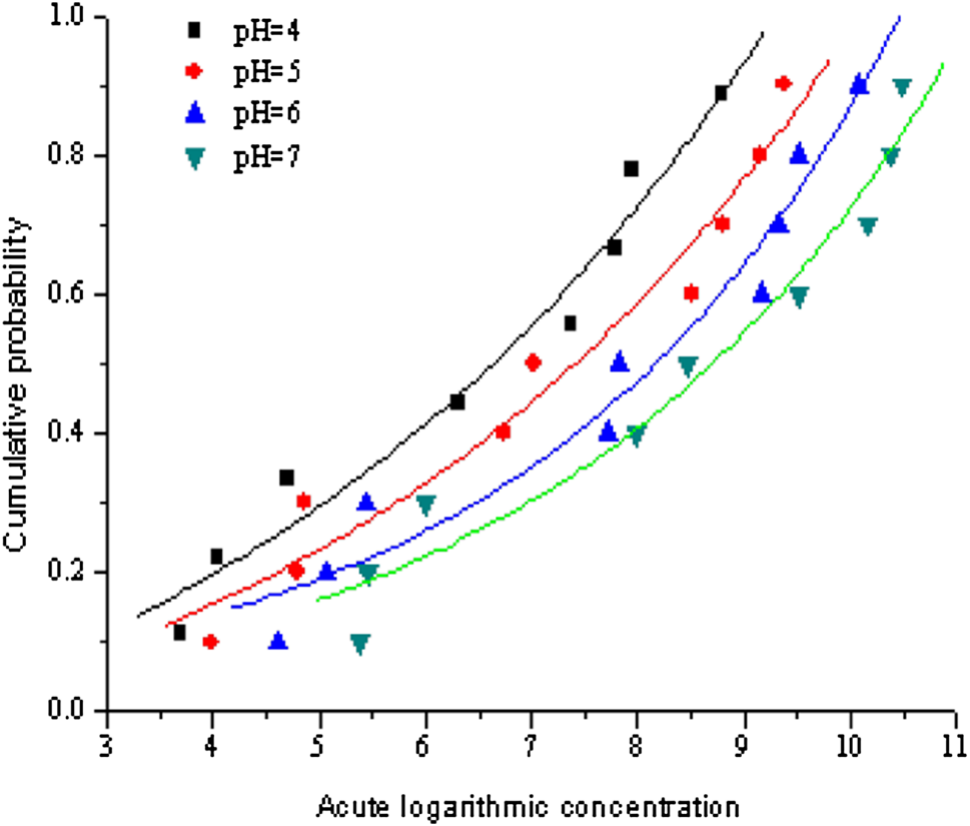
Comparisons of species sensitivity distributions for zinc under different pH values

SSD method could well show differences in the sensitivities of the suite of nine species to zinc at the different test pH values. Individual SSDs for zinc acute toxicity under each of the test pH values were derived (Fig. 2). SSD model-fitting trials indicated that all the toxicity data were best fitted by logistic models. The best-fitting models gave an R^2^ greater than 0.95. The results showed that the short-term hazardous concentrations (based on the 5th percentiles from each of the SSDs) 17.26 µg/L (pH = 4), 48.31 µg/L (pH = 5), 80.34 µg/L (pH = 6) and 230.6 µg/L (pH = 7), respectively. The short-term criteria for zinc have been previously derived to be 102.33 µg/L when pH was in the range of 6–9 (Liu et al. 2017). When pH was nearly 7 in a water environment, the water quality criteria of zinc was determined to be 115.3 µg/L using the SSD method, which was within 10% relative to the previous research. It was also found that zinc toxicity gradually increased with the constant decrease in the pH value in water. Hazardous concentration values would be reduced approximately two to three times with the reduction in pH values in the range of 4–7. This may be caused by the following reasons (Zvereva et al. 2003; Mazon et al. 1998): (1) the pH value in blood of aquatic animals changed greatly, and the oxygen carrying ability weakened, which resulted in hypoxia of aquatic animals. (2) It has a rather strong corrosive effect on the gill, skin and other tissues and organs of aquatic animals. (3) It has destructive effects on antioxidant enzymes and hydrolytic enzymes of the normal life of aquatic animals, resulting in metabolic disorder. (4) Peracid water would also cause a large number of bacteria and the reproduction of algae in water.

The current environmental quality standards for surface water in China are divided into five levels (GB 3838-2002) (Ministry of Environmental Protection of the People’s Republic of China 2002). Class I standard is the most stringent, and Class V the least stringent. The five levels of zinc standards are 0.05, 1, 1, 2, and 2 mg/L, respectively. Based on the measured concentrations of dissolved zinc in various aqueous solutions under different pH values, it could be found that when the pH was greater than 9, the measured concentration is far less than the nominal concentration, and it was difficult to reach the class II–V standards. Therefore, it could be easily found that the current water quality standards for zinc, such as the environmental quality standards for surface water and fisheries, were suitable when the pH was between 7.0 and 8.0. Due to the significant influence of pH on zinc toxicity, in the actual water bodies, the standards of zinc will over-protect or under-protect aquatic organisms determined by the actual acidic or alkaline water environment with a wide range of pH values. Accordingly, various environmental factors, such as pH, water hardness and organic matter, considered as an integrated chemical system, should be taken into consideration in the validation and revision of current standards for metals in Chinese waters in the near future.

## Electronic supplementary material

Below is the link to the electronic supplementary material.

Supplementary material 1 (DOC 160 KB)
