# Supplementary material for: Acute Toxicity and Hazardous Concentrations of Zinc to Native Freshwater Organisms Under Different pH Values in China
Source: Bull Environ Contam Toxicol. 2018 Sep 24;103(1):120–6. doi: 10.1007/s00128-018-2441-2 (PMC6647607; doi:10.1007/s00128-018-2441-2)
Supplement: Supplementary file 1 — Supplementary material 1 (DOC 160 KB) [file 128_2018_2441_MOESM1_ESM.doc]

[Supplementary](javascript:;) [materials](javascript:;)

Table S1 Concentrations of zinc under different pH values (mg/L)

| nominal | measured concentration (mg/L) | | | | | | | | |
| --- | --- | --- | --- | --- | --- | --- | --- | --- | --- |
| concentration | pH=3 | pH=4 | pH=5 | pH=6 | pH=7 | pH=8 | pH=9 | pH=10 | pH=11 |
| 0.025 | 0.025±0.001 | 0.026±0.001 | 0.027±0.001 | 0.025±0.001 | 0.023±0.001 | 0.024±0.001 | nd | nd | nd |
| 0.05 | 0.048±0.003 | 0.045±0.002 | 0.049±0.001 | 0.049±0.002 | 0.050±0.001 | 0.044±0.002 | nd | nd | nd |
| 0.1 | 0.09±0.011 | 0.09±0.011 | 0.098±0.019 | 0.120±0.012 | 0.106±0.002 | 0.106±0.002 | 0.11±0.001 | 0.01±0.001 | 0.01±0.001 |
| 0.2 | 0.21±0.021 | 0.19±0.012 | 0.21±0.022 | 0.204±0.003 | 0.20±0.011 | 0.22±0.014 | 0.20±0.011 | 0.01±0.013 | 0.03±0.011 |
| 0.4 | 0.41±0.011 | 0.41±0.012 | 0.41±0.011 | 0.41±0.011 | 0.40±0.012 | 0.40±0.021 | 0.30±0.011 | 0.04±0.012 | 0.03±0.013 |
| 0.8 | 0.81±0.012 | 0.78±0.011 | 0.80±0.011 | 0.82±0.015 | 0.81±0.014 | 0.80±0.012 | 0.57±0.013 | 0.24±0.013 | 0.07±0.013 |
| 1 | 1.12±0.011 | 1.04±0.012 | 1.02±0.012 | 1.01±0.013 | 1.07±0.010 | 1.09±0.011 | 0.68±0.012 | 0.36±0.012 | 0.09±0.013 |
| 1.6 | 1.67±0.022 | 1.58±0.014 | 1.59±0.021 | 1.58±0.013 | 1.57±0.021 | 1.63±0.015 | 0.43±0.013 | 0.53±0.012 | 0.11±0.013 |
| 2 | 2.01±0.013 | 2.13±0.023 | 2.09±0.021 | 2.08±0.021 | 2.06±0.022 | 2.02±0.021 | 0.76±0.011 | 0.60±0.012 | 0.22±0.012 |
| 3.2 | 3.19±0.034 | 3.34±0.045 | 3.21±0.041 | 3.20±0.041 | 3.25±0.042 | 2.18±0.022 | 0.81±0.012 | 0.81±0.012 | 0.18±0.012 |
| 4 | 4.05±0.021 | 4.31±0.039 | 4.05±0.031 | 4.03±0.031 | 4.08±0.031 | 2.29±0.027 | 0.84±0.014 | 0.69±0.012 | 0.21±0.012 |
| 6.4 | 6.29±0.063 | 6.31±0.057 | 6.38±0.063 | 6.33±0.062 | 6.35±0.071 | 2.64±0.018 | 0.73±0.013 | 0.56±0.012 | 0.31±0.012 |
| 8 | 8.07±0.047 | 8.10±0.041 | 8.06±0.048 | 8.02±0.073 | 8.09±0.072 | 1.89±0.016 | 0.67±0.012 | 0.55±0.012 | 0.46±0.012 |
| 16 | 15.79±0.28 | 15.06±0.21 | 16.03±0.14 | 16.08±0.141 | 16.02±0.081 | 1.91±0.073 | 0.36±0.013 | 0.26±0.014 | 0.12±0.011 |
| 32 | 31.28±0.28 | 32.04±0.14 | 32.05±0.28 | 32.09±0.21 | 31.89±0.212 | 2.18±0.142 | 0.27±0.013 | 0.10±0.012 | 0.03±0.012 |
| 64 | 63.79±0.61 | 64.27±0.35 | 63.81±0.35 | 64.05±0.28 | 63.04±0.215 | 1.78±0.071 | 0.31±0.012 | 0.29±0.012 | 0.06±0.011 |

“nd” indicates that the value and not be detected.
